# Deep fine-KNN classification of ovarian cancer subtypes using efficientNet-B0 extracted features: a comprehensive analysis

**DOI:** 10.1007/s00432-024-05879-z

**Published:** 2024-07-25

**Authors:** Santi Kumari Behera, Ashis Das, Prabira Kumar Sethy

**Affiliations:** 1 Department of Computer Science and Engineering, VSSUT, Burla, Odisha, 768018 India; 2 Department of Computer Science and Engineering, SUIIT, Sambalpur University, Burla, Odisha, 768019 India; 3 Present Address: Department of Electronics and Communication Engineering, Guru Ghasidas Vishwavidyalaya, Bilaspur, Chhattisgarh 495009 India; 4 Department of Electronics, Sambalpur University, Jyoti Vihar, Burla, Odisha, 768019 India

**Keywords:** Ovarian cancer, Deep learning, EfficientNetb0, KNN, Subtype ovarian cancer, Classification

## Abstract

This study presents a robust approach for the classification of ovarian cancer subtypes through the integration of deep learning and k-nearest neighbor (KNN) methods. The proposed model leverages the powerful feature extraction capabilities of EfficientNet-B0, utilizing its deep features for subsequent fine-grained classification using the fine-KNN approach. The UBC-OCEAN dataset, encompassing histopathological images of five distinct ovarian cancer subtypes, namely, high-grade serous carcinoma (HGSC), clear-cell ovarian carcinoma (CC), endometrioid carcinoma (EC), low-grade serous carcinoma (LGSC), and mucinous carcinoma (MC), served as the foundation for our investigation. With a dataset comprising 725 images, divided into 80% for training and 20% for testing, our model exhibits exceptional performance. Both the validation and testing phases achieved 100% accuracy, underscoring the efficacy of the proposed methodology. In addition, the area under the curve (AUC), a key metric for evaluating the model’s discriminative ability, demonstrated high performance across various subtypes, with AUC values of 0.94, 0.78, 0.69, 0.92, and 0.94 for MC. Furthermore, the positive likelihood ratios (LR^+^) were indicative of the model’s diagnostic utility, with notable values for each subtype: CC (27.294), EC (9.441), HGSC (12.588), LGSC (17.942), and MC (17.942). These findings demonstrate the effectiveness of the model in distinguishing between ovarian cancer subtypes, positioning it as a promising tool for diagnostic applications. The demonstrated accuracy, AUC values, and LR^+^ values underscore the potential of the model as a valuable diagnostic tool, contributing to the advancement of precision medicine in the field of ovarian cancer research.

## Introduction

Ovarian cancer is a formidable adversary within the spectrum of cancers of the female reproductive system, marked by its ominous distinction as the most lethal among its counterparts. The complexity of this disease is accentuated by its diverse subtypes, each of which is characterized by unique cellular morphologies, etiologies, molecular and genetic profiles, and clinical attributes. Despite being the eighth most common cancer in women worldwide and the fourth most common cancer in Indian women, ovarian cancer poses a significant challenge due to its asymptomatic nature in the early stages, leading to delayed detection and diagnosis. The World Health Organization (WHO) reported approximately 313,959 new cases and 207,252 deaths globally in 2020, underscoring the urgent need for effective diagnostic strategies (Chhikara et al. 2022).

Early diagnosis and treatment of ovarian cancer are pivotal for improving patient outcomes and enhancing the efficacy of therapeutic interventions. However, the asymptomatic nature of the disease in its initial stages often results in delayed detection, making it more challenging to treat at advanced stages, and is associated with lower survival rates. The five common subtypes, high-grade serous carcinoma, clear-cell ovarian carcinoma, endometrioid, low-grade serous, and mucinous carcinoma, together with various rare subtypes, collectively contribute to the intricate development of this formidable disease. The emergence of subtype-specific treatment approaches holds promise in the ongoing battle against ovarian cancer. Nevertheless, accurate subtype identification, which is crucial for unlocking the full potential of targeted therapies, currently relies on traditional diagnostic methods fraught with challenges, including interobserver disagreements and issues related to diagnostic reproducibility.

Efforts to combat ovarian cancer are gaining momentum, particularly with the integration of data science and deep learning. The analysis of histopathological images, a cornerstone in the diagnostic process, can be significantly enhanced through the application of deep learning models. Despite this potential, challenges persist, such as the necessity for substantial training data ideally sourced from a single diverse dataset. Overcoming technical, ethical, financial, and confidentiality constraints is paramount for unleashing the full potential of deep learning in revolutionizing ovarian cancer diagnosis.

In this rapidly evolving landscape, the convergence of data science and deep learning offers a promising avenue for improving the diagnosis and treatment of ovarian cancer. The power of advanced technology, particularly in the analysis of histopathological images, is key to more accurate and efficient identification of ovarian cancer subtypes. However, addressing the training data challenges, ethical considerations, financial constraints, and confidentiality issues is essential for harnessing the full potential of deep learning in this critical context. By overcoming these hurdles, we can pave the way for a future where early diagnosis becomes more accessible and targeted therapies can be optimized, ultimately improving patient outcomes and advancing the fight against ovarian cancer.

The major contributions of this study are as follows.

- Deep learning methodologies were incorporated to harness the powerful feature extraction capabilities of EfficientNet-B0, enhancing the ability of the model to capture intricate patterns in histopathological images.
- Five distinct ovarian cancer subtypes were assessed in this study—HGSC, CC, EC, LGSC, and MC—contributing to a thorough understanding of the model’s applicability.
- The present quantitative metrics, such as the area under the curve (AUC) and LR + for each subtype, provided a standardized and measurable assessment of the model’s diagnostic performance.
- It achieved 100% accuracy in both the training and testing phases, showing the model’s efficacy in subtype classification.
- This model is a promising tool for diagnostic applications, contributing to the progress of precision medicine in ovarian cancer research by facilitating targeted and effective treatments.

The structure of the article is outlined as follows. Sect. “Literature review” contains the literature review. In Sect. “Materials and methodology”, materials and methodology are elaborated. Sect. “Results and discussion” presents the results and discussion. Finally, the article concludes in Sect. “Conclusion”.

## Literature review

Numerous research endeavors have significantly advanced our understanding of ovarian cancer by exploring diverse methodologies, ranging from deep learning applications and innovative imaging techniques to multimodal analyses and novel algorithmic architectures. This literature review synthesizes collective contributions, each offering a unique perspective and transformative insights that collectively propel the field forward. The amalgamation of these diverse studies provides a comprehensive picture of the evolving landscape of ovarian cancer research, showcasing the ongoing quest for more accurate diagnostics and effective treatment strategies.

The groundbreaking work conducted by Hu et al. (2023) demonstrated promising results, showing that deep learning methods can effectively segment EOC. The performances of different algorithms, including U-Net, DeepLabv3, U-Net +  + , PSPNet, TransUnet, and Swin-Unet, were evaluated using metrics such as the Dice similarity coefficient (DSC), Hausdorff distance (HD), average symmetric surface distance (ASSD), precision, and recall (Hu et al. 2023).

Ziyambe et al. (2023) introduced an innovative convolutional neural network (CNN) algorithm to address these limitations and enhance the prediction and diagnosis of ovarian cancer. The CNN was trained on a histopathological image dataset, which was partitioned into training and validation subsets and underwent augmentation before the training process. Remarkably, the model achieved an accuracy of 94%, correctly identifying 95.12% of cancerous cases and accurately classifying 93.02% of healthy cells (Ziyambe et al. 2023).

Gajjela et al. (2023) introduced a novel technique, optical photothermal infrared (O-PTIR) imaging, as a label-free and automated method for the histological recognition of ovarian tissue subtypes. Mid-infrared spectroscopic imaging (MIRSI) was used, offering a 10 × improvement in spatial resolution compared to previous instruments. This approach has been used for traditional histopathological identification of ovarian cancer via time-consuming staining and subjective pattern recognition. This technique allows subcellular spectroscopic analysis at crucial fingerprint wavelengths, thus enhancing the identification of ovarian cell subtypes with a notable classification accuracy of 0.98. This study included a robust analysis of 78 patient samples comprising over 60 million data points. Significantly, subcellular resolution using only five wavenumbers surpasses diffraction-limited techniques employing up to 235 wavenumbers (Gajjela et al. 2023).

Wang et al. (2023) introduced MMDAE-HGSOC, a multimodal deep autoencoder learning approach. It integrates miRNA expression, DNA methylation, copy number variation (CNV), and mRNA expression data to construct a comprehensive multiomics feature space. A multimodal deep autoencoder network was employed to learn high-level feature representations, and a novel superposition LASSO (S-LASSO) regression algorithm was proposed for the precise identification of genes associated with HGSOC molecular subtypes. The experimental results demonstrate the superiority of MMDAE-HGSOC over existing classification methods. Additionally, this study investigated the enrichment of gene ontology (GO) terms and biological pathways associated with the selected significant genes, offering valuable insights into the underlying mechanisms of HGSOC (Wang et al. 2023).

Kodipalli et al. (2023) focused on innovating a novel convolutional neural network (CNN) architecture and compared its performance against established models, including those recognized in the ImageNet Large Scale Visual Recognition Challenge (ILSVRC). This study utilized high-quality ovarian CT images, employing cloud services such as the Google Cloud Platform for training and evaluation. The proposed CNN variant achieved an impressive accuracy of 97.53%, surpassing the performance of existing architectures and demonstrating its efficacy in classifying ovarian tumors (Kodipalli et al. 2023).

Wu et al. (2023) studied 1142 ultrasound images from 328 patients (from January 2019 to June 2021) to assess the performance of deep convolutional neural networks (DCNNs) in distinguishing various histologic types of ovarian tumors. Task 1 involved classifying benign and high-grade serous carcinomas in the original images, while Task 2 focused on segmented images. Transfer learning was applied to six pretrained DCNNs. The ResNext50 model demonstrated superior performance, achieving 95.2% accuracy in classifying seven ovarian tumor types with notable sensitivity and specificity. Overall, the DCNN has emerged as a promising tool for detailed ovarian tumor classification in ultrasound images, offering valuable computer-aided diagnostic insights (Wu et al. 2023).

Bergstrom et al. (2023) proposed a novel approach that addresses the challenge of detecting homologous recombination deficiencies (HRDs) in breast and ovarian cancers, which is crucial for guiding treatment decisions involving platinum-based therapies and PARP inhibitors. By utilizing deep learning on routinely obtained hematoxylin and eosin-stained histopathological slides, this method accurately predicted genomically derived HRD scores. External validation of breast cancer cohorts demonstrated its efficacy in predicting patient responses to platinum treatment, whereas transfer learning extended its clinical utility to high-grade ovarian tumors. Notably, this deep learning model surpasses existing genomic HRD biomarkers, offering a valuable alternative for HRD detection, particularly in medically underserved populations (Bergstrom et al. 2023).

Zhang et al. (2019) proposed an innovative image diagnosis system for ovarian cyst classification using color ultrasound images, addressing the challenge of accurately distinguishing between benign and malignant nodules. Our approach combines high-level features from a fine-tuned GoogLeNet neural network with low-level rotation-invariant uniform local binary-pattern (ULBP) features. After enhancing the ultrasound images, we extracted ULBP features to capture texture descriptors and normalized and concatenated them with deep features to form fusion features. These fusion features were then input into a cost-sensitive random forest classifier for accurate classification. The integration of high-level semantic context and low-level texture patterns effectively discerns the differences between malignant and benign ovarian cysts, thereby reducing unnecessary medical procedures and associated costs (Zhang et al. 2019). The details of the methodology adapted to the performance are presented in Table 1.

**Table 1:** Summary of Key Research Contributions in Ovarian Cancer: Methods, Datasets, and Achievements

Reference	Adapted methodology	Dataset used	Remark
Hu et al. (2023)	Deep learning (U-Net, DeepLabv3, U-Net + + , PSPNet, TransUnet, Swin-Unet)	Not specified in the provided information	Promising results in EOC segmentation
Ziyambe et al. (2023)	Convolutional neural network (CNN)	Histopathological image dataset	94% accuracy in predicting and diagnosing ovarian cancer
Gajjela et al. (2023)	Optical photothermal infrared (O-PTIR) imaging	78 patient samples	0.98 classification accuracy, subcellular resolution surpassing traditional techniques
Wang et al. (2023)	Multimodal deep autoencoder learning	miRNA expression, DNA methylation, CNV, mRNA expression data	Superiority over existing classification methods, insights into HGSOC mechanisms
Kodipalli et al. (2023)	Novel CNN architecture	High-quality ovarian CT scan images	97.53% accuracy in classifying ovarian tumors
Wu et al. (2023)	Deep convolutional neural networks (DCNN)	1142 ultrasound images from 328 patients	ResNet50 model achieved 95.2% accuracy in classifying ovarian tumor types
Bergstrom et al. (2023)	Deep learning on hematoxylin and eosin-stained histopathological slides	Not specified in the provided information	Superiority in HRD detection over existing genomic biomarkers
Zhang et al. (2019)	Image diagnosis system	Color ultrasound images	Fusion of high-level and low-level features for accurate ovarian cyst classification

According to a comprehensive literature review, only Ziyambe et al. (2023) specifically studied ovarian cancer by utilizing histopathological images, albeit with limited two-way classification. Despite the significant advancements in ovarian cancer research through various deep learning applications and multimodal analyses, a research gap persists in the integration of advanced feature extraction methods and traditional classification techniques for enhanced diagnostic accuracy. While studies have demonstrated promising results using individual methodologies, there is a lack of comprehensive approaches that combine the powerful feature extraction capabilities of deep learning models like EfficientNet-B0 with traditional classifiers such as k-nearest neighbor (KNN). This integration could potentially yield more precise and reliable diagnostic tools, addressing the current limitations of interobserver variability and diagnostic reproducibility in ovarian cancer subtype classification. This observation underscores the significant gap in research on the more nuanced exploration of subtype classification in ovarian cancer. Consequently, the need for further investigation into subtype classification has emerged as a promising and largely unexplored area within the field, presenting a valuable avenue for future research.

## Materials and methodology

This section presents detailed information on the datasets used and methodologies adopted in this study.

### Dataset

**Fig. 1:**
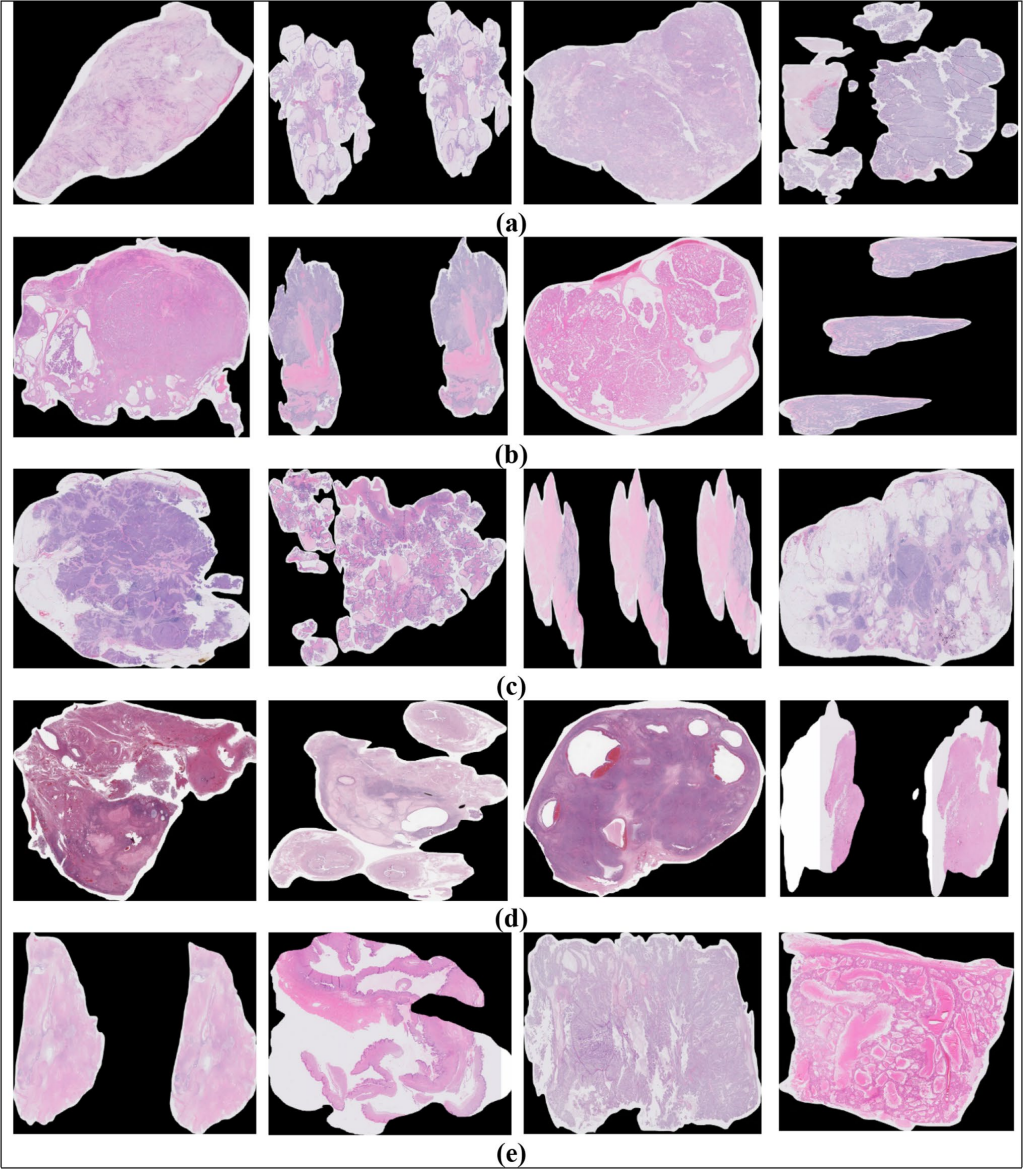
Samples of ovarian cancer subtypes histopathological images **a** CC **b** EC **c** HGSC **d** LGSC **e** MC

The UBC-OCEAN (Bashashati et al. 2023) dataset is a valuable resource in the field of histopathological research, providing a diverse collection of high-resolution images for two distinct categories of ovarian cancer: whole slide images (WSIs) and tissue microarrays (TMAs). This dataset contains 505 images. The images were resized to 512 × 512 pixels. Details of the dataset are presented in Table 2. Histopathological images of the ovarian subtypes are shown in Fig. 1.

**Table 2:** Details of Dataset before and after augmentation

Dataset	Name of the class	No. of images present in the class before augmentation	No. of images present in the class after augmentation	Image type	Size of the folder before compression (before augmentation)	Size of the folder after compression (after augmentation)
UBC-OCEAN	EC	118	145	All the images are in the form of PNG	185.5 GB	65.8 MB
	CC	90	145		141.4 GB	46.7 MB
	HGSC	216	145		339.5 GB	52.9 MB
	LGSC	40	145		62.8 GB	55.6 MB
	MC	41	145		64.4 GB	56.3 MB
Total		505	725		794.01 GB	277.3 MB

### Methodology

The methodology employed in this research centers on leveraging the deep features of EfficientNet-B0 for the classification of ovarian cancer subtypes. EfficientNet-B0 is characterized by a streamlined architecture that incorporates MobileNetV2-like bottleneck blocks, specifically MBConv1 and MBConv6. These blocks play a crucial role in feature extraction and offer an effective balance between model complexity and accuracy.

To tailor the model for subtype classification, a fine-KNN approach was integrated, involving replacement of the last layer, representing the fully connected layer, with the fine-KNN mechanism. This modification facilitates the nuanced classification of histopathological images into five distinct ovarian cancer subtypes: high-grade serous carcinoma (HGSC), clear-cell ovarian carcinoma (CC), endometrioid carcinoma (EC), low-grade serous carcinoma (LGSC), and mucinous carcinoma (MC).

**Fig. 2:**
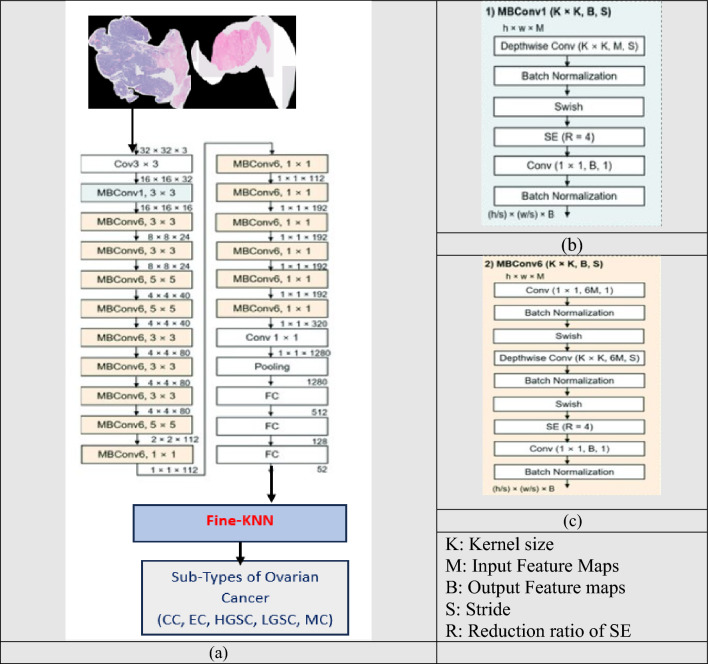
Architecture Diagram: Integration of EfficientNetB0 and Fine-KNN for Ovarian Cancer Subtype Classification **a** Main architecture **b** MBConv1 **c** MBConv6

The architecture of EfficientNet-B0 warrants closer examination, particularly regarding the characteristics of its MBConv1 and MBConv6 blocks. MBConv1 employs a 3 × 3 depthwise separable convolution, followed by batch normalization and a swish activation function. In contrast, MBConv6 incorporates a 3 × 3 depthwise separable convolution with a larger expansion ratio, enhancing the representational power of the model. The architectural details are shown in Fig. 2.

The performance evaluation of the model involves key metrics, such as accuracy, which provide an overarching measure of correct classification. Additionally, the area under the curve (AUC) values were computed to assess the discriminative ability of the model across different subtypes, providing a more comprehensive understanding of its diagnostic efficacy.

For a more nuanced evaluation, the true positive rate (TPR) and false negative rate (FNR) were calculated for each subtype. This in-depth analysis provides insights into the model’s ability to correctly identify instances of each subtype and its potential to minimize false negatives.

The diagnostic reliability of the model was further evaluated by calculating the positive likelihood ratios (LRs). This metric offers insights into the ability of the model to provide diagnostic certainty for each subtype, adding a layer of assurance to the classification results.

In summary, this methodology integrates the robust feature extraction capabilities of EfficientNet-B0 with the fine-KNN mechanism, presenting a comprehensive approach for the subtype classification of ovarian cancer histopathological images. The subsequent evaluation metrics ensured a thorough analysis of the model’s diagnostic process and its potential contribution to precision medicine in the realm of ovarian cancer research.

## Results and discussion

**Fig. 3:**
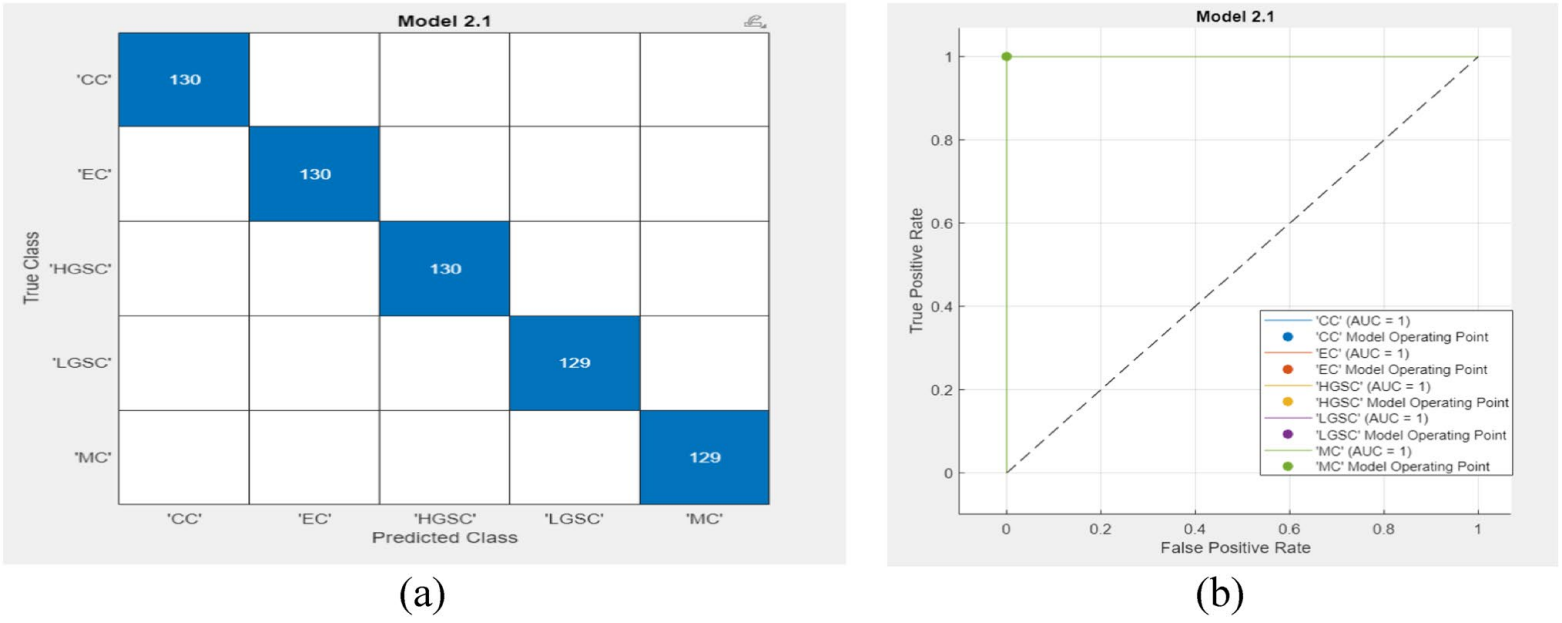
Validation results of Efficientnetb0 with Fine KNN **a** confusion matrix **b** AUC

**Fig. 4:**
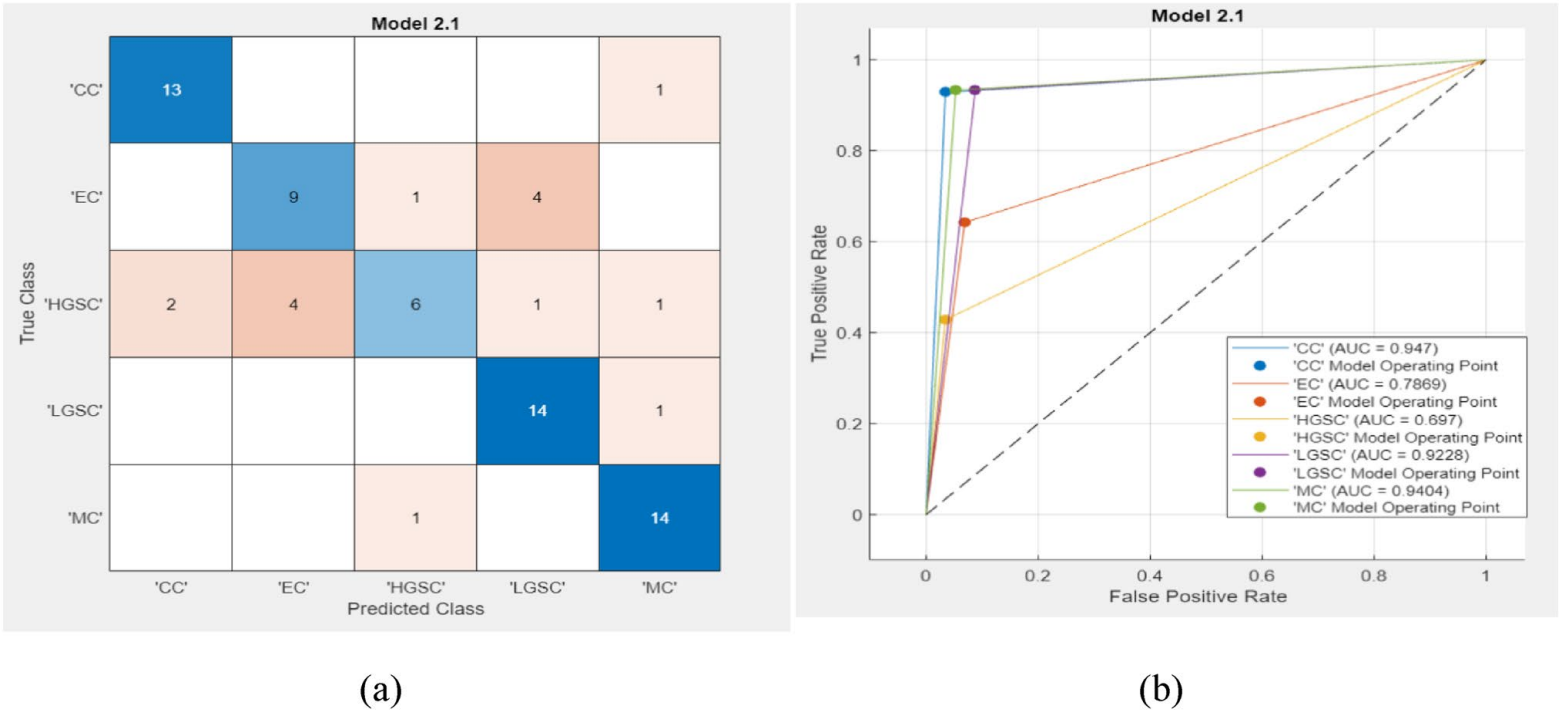
Test results of Efficientnetb0 with Fine KNN **a** confusion matrix **b** AUC

In our study, utilizing the UBC-OCEAN dataset of 725 histopathological images representing five ovarian cancer subtypes, our model showed robust performance. The dataset was thoughtfully split, with 80% for training and 20% for testing, ensuring a thorough evaluation. Our model is implemented on an HP laptop with an 11th generation processor, 16 GB of RAM, and an NVIDIA 3070 GPU and operates efficiently on MATLAB 2022a. With an initial learning rate of 0.001, a mini-batch size of 32, and the ADAM optimizer, our model leverages the fine-KNN approach for nuanced subtype classification. The results revealed high accuracy during testing, confirming the model’s ability to distinguish between HGSC, CC, EC, LGSC, and MC. The individual subtype metrics provide deeper insights. The performances of the models on the validation and test data are shown in Figs. 3 and 4, respectively.

Based on the insights gleaned from Figs. 3 and 4, our proposed methodology achieves remarkable results. Both the validation and test accuracies reached 100%, confirming the effectiveness of our approach. Furthermore, the area under the curve (AUC) is a crucial metric for assessing a model’s discriminative ability, which reinforces its high performance. Notably, during testing, the AUC values were noteworthy for various subtypes, with values of 0.94 for CC, 0.78 for EC, 0.69 for HGSC, 0.92 for LGSC, and 0.94 for MC. It notices that, discrepancy between the original sample sizes and predictive performances for EC and HGSC subtypes even if make balanced using augmentation. While EC and HGSC indeed had the highest sample sizes, several factors could explain the lower predictive performance. Firstly, the inherent biological heterogeneity and overlapping histopathological features of these subtypes might have contributed to the classification challenges, causing the model to struggle in distinguishing them from other subtypes. Additionally, the variability within the EC and HGSC subtypes themselves, which can exhibit a broad spectrum of morphological patterns, may have led to a reduced ability of the model to generalize well to the test data. Furthermore, despite the large sample sizes, there may still be an imbalance in the representation of certain histopathological characteristics within these subtypes, impacting the model’s training process. To address these issues, future work could focus on incorporating advanced data augmentation techniques and exploring additional features or integrating multimodal data to enhance the model’s ability to capture the distinctive characteristics of EC and HGSC subtypes. This will help improve the overall predictive performance and robustness of the model.

Furthermore, the model was evaluated for each class of ovarian cancer, as shown in Tables 3, 4, and 5.

**Table 3:** TPR, FNR, PPV, and FDR of each subtype of Ovarian Cancer in Validation

Sub-types of ovarian cancer	TPR (%)	FNR (%)	PPV (%)	FDR (%)
CC	100	0	100	0
EC	100	0	100	0
HGSC	100	0	100	0
LGSC	100	0	100	0
MC	100	0	100	0

**Table 4:** TPR, FNR, PPV, and FDR of each subtype of Ovarian Cancer in Test

Sub-types of ovarian cancer	TPR (%)	FNR (%)	PPV (%)	FDR (%)
CC	92.9	7.1	86.7	13.3
EC	64.3	35.7	69.2	30.8
HGSC	42.9	57.1	75.0	25.0
LGSC	93.3	6.7	73.7	26.3
MC	93.3	6.7	82.4	17.6

**Table 5:** Calculation of LR^+^ values based on TPR and FPR values for the test case

Sub-types of ovarian cancer	TPR	FPR	${LR}^{+}=\frac{TPR}{FPR}$
CC	0.928	0.034	27.294
EC	0.642	0.068	9.441
HGSC	0.428	0.034	12.588
LGSC	0.933	0.052	17.942
MC	0.933	0.052	17.942

As shown in Table 3, the validation dataset demonstrated outstanding performance across all subtypes, with a perfect true positive rate (TPR) of 100% for clear cell (CC), endometrioid (EC), high-grade serous (HGSC), low-grade serous (LGSC), and mucinous (MC) ovarian cancers. The false negative rate (FNR) was also consistently zero, highlighting the ability of the model to correctly identify instances of each subtype. Furthermore, the positive predictive value (PPV) and false discovery rate (FDR) both reached 100%, underscoring the precision and reliability of the classification.

From Table 4, it can be observed that while the performance in the test dataset remained strong, some variations were observed. Notably, the sensitivity (TPR) for the EC, HGSC, and LGSC subtypes decreased, with the lowest value observed in HGSC at 42.9%. The corresponding increase in the FNR suggests potential challenges in correctly identifying these subtypes. Despite this, the overall performance remained robust, with TPR values exceeding 90% for CC, LGSC, and MC. The PPV and FDR values provide insights into the precision of the model in the test dataset. The PPVs for EC and HGSC indicated the potential for false positives, with values of 69.2 and 75.0%, respectively. However, the FDR is generally low across all subtypes, demonstrating reliable control over false positives. Table 5 shows the values of the likelihood ratio positive (LR +), which reinforce the overall diagnostic performance. A higher LR + indicates a more reliable positive test result. Notably, the CC, LGSC, and MC subtypes exhibited particularly high LR + values, suggesting their strong ability to correctly identify these ovarian cancer subtypes.

The validation dataset shows the exemplary performance of the classification model, indicating its ability to accurately identify ovarian cancer subtypes. The minor discrepancies observed in the test dataset may be attributed to variations in the data distribution, emphasizing the importance of robust model validation. The LR + values provide additional context, indicating the strength of the model in providing reliable positive results. The higher LR + values for the CC, LGSC, and MC subtypes suggested that the model accurately identified these specific ovarian cancer subtypes.

Our model, which leverages the UBC-OCEAN dataset and combines EfficientNet-B0 with the fine-KNN approach, demonstrated robust performance with a 100% accuracy rate during both validation and testing phases, indicating its efficacy in accurately classifying ovarian cancer subtypes. The AUC values, a critical metric for assessing the model’s discriminative ability, were particularly high for CC, LGSC, and MC subtypes, underscoring the model’s strong performance. The validation dataset showed a perfect true positive rate (TPR) across all subtypes, while the test dataset revealed some variability, especially for EC and HGSC, highlighting areas for further refinement. Despite these variations, the overall performance remained robust, with high PPV and low FDR values. The likelihood ratio positive (LR +) values further confirmed the model’s reliability in providing accurate positive results, particularly for CC, LGSC, and MC subtypes. These findings collectively demonstrate the model’s potential as a highly accurate and reliable diagnostic tool, contributing significantly to the advancement of precision medicine in ovarian cancer diagnosis.

## Conclusion

This research introduces a robust methodology for ovarian cancer subtype classification that integrates deep learning and k-nearest neighbor (KNN) techniques. The model, built on EfficientNet-B0 with fine-KNN, demonstrated an outstanding accuracy of 100% across the clear cell (CC), endometrioid (EC), high-grade serous (HGSC), low-grade serous (LGSC), and mucinous (MC) subtypes during both the validation and test phases. The high area under the curve (AUC) values during testing further underscore the model’s discriminative ability. The positive likelihood ratio (LR +) values emphasize its diagnostic utility, particularly for CC, LGSC, and MC. Despite these achievements, future research should explore data augmentation, multimodal data integration, and interpretability to enhance generalizability, transparency, and clinical applicability. External validation and prospective clinical studies are crucial steps toward validating the model’s real-world performance and facilitating its integration into diagnostic workflows, thereby contributing to the advancement of precision medicine in ovarian cancer research.

## Data Availability

The available data and materials are available upon reasonable request.
